# SIRT6 attenuates LPS‐induced inflammation and apoptosis of lung epithelial cells in acute lung injury through ACE2/STAT3/PIM1 signaling

**DOI:** 10.1002/iid3.809

**Published:** 2023-03-17

**Authors:** Juan Yang, Xing Chen

**Affiliations:** ^1^ Department of Pediatric, Shandong Provincial Hospital Shandong University Jinan Shandong China

**Keywords:** ACE2/STAT3/PIM1 signaling, acute lung injury, apoptosis, inflammation, SIRT6

## Abstract

**Background:**

Acute lung injury (ALI) is a severe and fatal respiratory disease. SIRT6 exerts pivotal activities in the process of lung diseases, but whether SIRT6 impacts ALI has not been covered.

**Methods:**

Lentivirus recombinant expressing vector SIRT6 gene (Lent‐SIRT6) was constructed in mice, and there were control, lipopolysaccharide (LPS), LPS + Vehicle, and LPS + Lent SIRT6 groups. RT‐qPCR and western blot detected SIRT6 expression in lung tissues. HE staining observed pathological alternations in lung tissues. Wet‐to‐dry ratio of the lungs was then measured. The cell count of bronchoalveolar lavage fluid (BALF) was evaluated. Serum inflammation was examined with enzyme‐linked immunosorbent assay, terminal deoxynucleotidyl transferase dUTP nick end labeling (TUNEL), and western blot were to measure apoptosis. Western blot tested the expression of ACE2/STAT3/PIM1 signaling‐associated factors. At the cellular level, LPS was used to induce lung epithelial cells BEAS‐2B to establish cell injury models. SIRT6 was overexpressed and ACE2 expression was inhibited by cell transfection, and the mechanism of SIRT6 in LPS‐induced lung injury model was further explored by Cell Counting Kit‐8 (CCK‐8), western blot, quantitative reverse‐transcription polymerase chain reaction, TUNEL, and other techniques.

**Results:**

The results of animal experiments showed that SIRT6 overexpression could reduce LPS‐induced lung pathological injury, pulmonary edema, and BALF cell ratio and attenuate LPS‐induced inflammatory response and cell apoptosis. In the above process, ACE2, STAT3, p‐STAT3, and PIM1 expression were affected. In cell experiments, SIRT6 expression was reduced in LPS‐induced BEAS‐2B cells. Inhibition of ACE2 expression could reverse the inhibitory effect of SIRT6 overexpression on ACE2/STAT3/PIM1 pathway, and cellular inflammatory response and apoptosis.

**Conclusion:**

SIRT6 eased LPS‐evoked inflammation and apoptosis of lung epithelial cells in ALI through ACE2/STAT3/PIM1 signaling.

## Graphical abstract

Acute lung injury (ALI) is a severe and fatal respiratory disease. SIRT6 exerts pivotal activities in the process of lung diseases, but whether SIRT6 impacts ALI has not been covered. In this study, we found that SIRT6 eased lipopolysaccharide‐evoked inflammation and apoptosis of lung epithelial cells in ALI through ACE2/STAT3/PIM1 signaling.

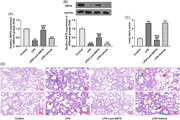

## 1 INTRODUCTION

Acute lung injury (ALI) is a clinical syndrome of acute respiratory failure caused by various intrapulmonary and extrapulmonary pathogenic factors.^1^ ALI is a severe respiratory disease featured by uncontrolled oxidative stress, pulmonary edema, inflammation, neutrophil infiltration, and so forth.^2^ Currently, the fatality rate of ALI remains 40%–60%.^3^ Therefore, it is very important to actively seek effective therapy for ALI.

Seven sirtuin subtypes (SIRT1–SIRT7) widely expressed in mammalian genomes play important roles in various physiological processes such as cell growth and apoptosis.^4^ Recent studies have confirmed that SIRT6 plays a key regulatory role in gene transcription, metabolism, maintenance of genomic stability, and telomere integrity.^5^, ^6^ A previous study has shown that SIRT6 expression is decreased in sepsis‐induced lung injury. Overexpression of SIRT6 can alleviate lipopolysaccharide (LPS)‐induced injury of vascular endothelial cells.^7^ A study has shown that SIRT6 has a protective effect on radiation‐induced lung injury.^8^ In addition, a number of studies have reported that SIRT1 has a protective effect on various types of lung injury, LPS‐induced lung injury is also included.^9^, ^10^, ^11^, ^12^ However, whether SIRT6 can play a role in LPS‐induced ALI is unknown.

A recent study has shown that SIRT6 has a protective effect on cholesterol crystal‐induced endothelial dysfunction by regulating ACE2 expression.^13^ SIRT6 can regulate ACE2 expression in rat aortic membrane fibroblasts.^14^ These results have implied that ACE2 expression can be modulated by SIRT6. ACE2 is the binding receptor of the severe acute respiratory syndrome coronavirus 2, so activation of ACE2 has an important protective effect on lung injury.^15^ Studies have shown that ACE2 can relieve lung injury caused by pulmonary hypertension by inhibiting downstream STAT3 signaling pathway to exert antioxidant activity.^16^, ^17^ Moreover, studies have clearly demonstrated that inhibition of STAT3 expression can significantly reduce LPS‐induced ALI.^18^, ^19^ KEGG website (http://www.kegg.jp) predicted that STAT3 activation could activate the expression of downstream target gene PIM1. Studies have shown that PIM1 inhibition can alleviate ALI.^20^, ^21^

Therefore, in this article, we mainly discuss the role of SIRT6 in ALI and the mechanism of SIRT6 in lung injury through the regulation of ACE2/STAT3/PIM1 signal.

## 2 MATERIAL AND METHODS

### 2.1 Animal grouping

Mice (4–6 week old, 20–30 g) were grouped into control (10 mL/kg saline by i.p. injection), LPS (10 mg/kg LPS by i.p. injection), LPS + vehicle and LPS + Lent‐SIRT6 groups (*n* = 6) at random. LPS + Lent‐SIRT6: lentivirus recombinant expressing vector SIRT6 gene (Lent‐SIRT6) was constructed by the company (Shanghai Genechem. Co., Ltd.) and a dose of 4 × 10^7^ (in 50 µL) was administered intranasally into the mice for 48 h, followed by LPS exposure (10 ml/kg). LPS + vehicle: the vehicle lentivirus recombinant protein vector was constructed by the company (Shanghai Genechem. Co., Ltd.) and 50 µL intranasal injection was executed for 48 h, followed by LPS exposure (10 mL/kg). The experimental protocols were approved by Shandong Provincial Hospital, Cheeloo College of Medicine, Shandong University (approve number: NO.2022‐016). All experimental processes were conducted in accordance with institutional animal guidelines and the ARRIVE guidelines.

### 2.2 Quantitative reverse‐transcription polymerase chain reaction (RT‐qPCR)

RNA isolation was performed from lung tissues using an Eastep Super Total RNA Extraction Kit. cDNAs were produced using Prime Script RT reagent Kit (Beijing Takara Biotechnology Co., Ltd.). The expression levels of mRNAs were quantified with the Bio‐Rad iCyCler real‐time PCR7500 system with SYBR Green technology. The gene expression was quantitatively analyzed by the method of 2^−ΔΔCq^.^22^ The primer sequences were as follows: mice SIRT6 forward: 5′‐ATGTCGGTGAATTATGCAGCA‐3′, reverse: 5′‐GCTGGAGGACTGCCACATTA‐3′; mice GAPDH forward: 5′‐AGGTCGGTGTGAACGGATTTG‐3′, reverse: 5′‐AGGTCGGTGTGAACGGATTTG‐3′; human ACE2 forward: 5′‐TGGGACTCTGCCATTTACTTAC‐3′, reverse: 5′‐CCCAACTATCTCTCGCTTCATC‐3′; humanβ‐actin forward: 5′‐CGGGAAATCGTGCGTGAC‐3′, reverse: 5′‐CAGGAAGGAAGGCTGGAAG‐3′.

### 2.3 Western blot

Bicinchoninic acid (BCA) kit (Beyotime) was to confirm protein concentration following isolation of proteins from cells utilizing RIPA buffer (Beyotime) in light of the manufacturer's instructions. Then polyvinylidene fluoride membranes were to transfer 30 μg of protein per well loaded into 10% sodium dodecyl sulfate‐polyacrylamide gel electrophoresis gel. Primary antibodies and horseradish peroxidase‐conjugated secondary antibody (Protein Tech Group) at a dilution of 1:5000 were supplemented to the membranes impeded with 5% of skim milk powder respectively at 4°C for overnight and for 1 h at room temperature. Animal‐related proteins were developed by using the film developer. For cellular‐related proteins, ECL substrates (Thermo Pierce) were used to protein development. And ImageJ (Version146) was to develop and analyze the density of immunoblotting bands.

### 2.4 Histopathological examination (HE) staining

Following immobilization with 10% formalin solution for 24 h at 4°C, hematoxylin and eosin (H&E) were to stain paraffin‐embedded tissues which were cut into 5 μm‐thick sections. The lung injury was assessed by histological examination. According to the histological injury parameters, including lung tissue edema, alveolar septal cell exudation, congestion, and alveolar hemorrhage, lung tissue injury was divided into four grades: normal (0 points), mild (1 points), moderate (2 points), and severe (3 points), and semi‐quantitative analysis of the injury was performed. The total lung injury score was calculated as the summation of individual scores of each item.

### 2.5 Measurement of wet‐to‐dry ratio (W/D) of the lungs

Following dissection in mice, “wet” weight of the right lungs was acquired. Then “dry” weight was acquired after the lungs were dried at 60°C for 48 h. The extent of pulmonary edema was evaluated using the Wet/Dry ratio.

### 2.6 Giemsa staining

The 0.3 mL precooled saline was to irrigate the lungs of mice for three times and the harvested BALF was centrifugated at 2000 r/min for 5 min. Bronchoalveolar lavage fluid (BALF) was applied to the slide, which then dried naturally. About 2–3 drops of Giemsa staining solution covered the entire specimen smear for 1–2 min. An equal amount of phosphate buffer (pH 6.4) was added to the slide. The slide was gently shook and thoroughly mixed with Giemsa staining solution for 3–5 min. Finally, the slides were washed, dried, stained, and examined for photo analysis. Then cell counter was to examine total cells, neutrophils, and macrophages proportion in BALF.

### 2.7 Enzyme‐linked immunosorbent assay (ELISA)

Interleukin (IL)‐1β, tumor necrosis factor‐α (TNF‐α), and IL‐6 levels were measured in BALF collected from another three mice of each group using ELISA kits (Nanjing Jiancheng Bioengineering Institute) according to the manufacturer's protocols.

### 2.8 Terminal deoxynucleotidyl transferase dUTP nick end labeling (TUNEL) staining

The lung cells were prepared, and cell apoptosis was measured by the Apoptosis Fluorescein Detection Kit (Millipore). The cells were fixed in 4% paraformaldehyde for 5 min. Terminal deoxyribonucleotidyl transferase and deoxyuridine triphosphate was to cultivate 4% paraformaldehyde‐immobilized cells. Nuclear staining was performed with DAPI (Invitrogen). Image‐Pro Plus 6.0 software was to analyze the results captured by a fluorescence microscope.

### 2.9 Cell culture

Dulbecco's modified Eagle's medium (DMEM) (Gibco) was to culture human alveolar epithelial cells BEAS‐2B procured from American Type Culture Collection (ATCC) with 10% fetal bovine serum (Gibco), 1% penicillin‐streptomycin at 37°C with 5% CO_2_. Different doses of LPS (100, 200, 250, 300, 350, 400, and 500 μg/mL) were to treat BEAS‐2B cells for 24 h.

### 2.10 Cell transfection

SIRT6 overexpression plasmid and ACE2 interference plasmids (siRNA‐ACE2‐1/2/3) and their blank control were constructed by Shanghai Genomics Chemistry Co., Ltd.

### 2.11 Cell Counting Kit‐8 (CCK8) assay

After indicated treatment of a total of 5000 cells seeded into 96‐well plates accordingly, the medium was discarded, and each well of the plate was supplemented with 100 µL fresh medium with 10% CCK8 solution (Dojindo) for 2 h. A microplate reader (Thermo Fisher) was to measure OD450 nm value.

### 2.12 Statistical analysis

Data analyzed by SPSS 22.0 software were denoted as mean ± standard deviation (SD). Unpaired Student's *t*‐test or one‐way analysis of variance (ANOVA) analysis followed by Tukey post hoc test was to compare group differences. It was regarded to possess statistical significance when *p* value < .05. The experiment was repeated at least three times.

## 3 RESULTS

### 3.1 Overexpression of SIRT6 alleviated lung pathological injury in LPS‐induced mice

**Figure 1:**
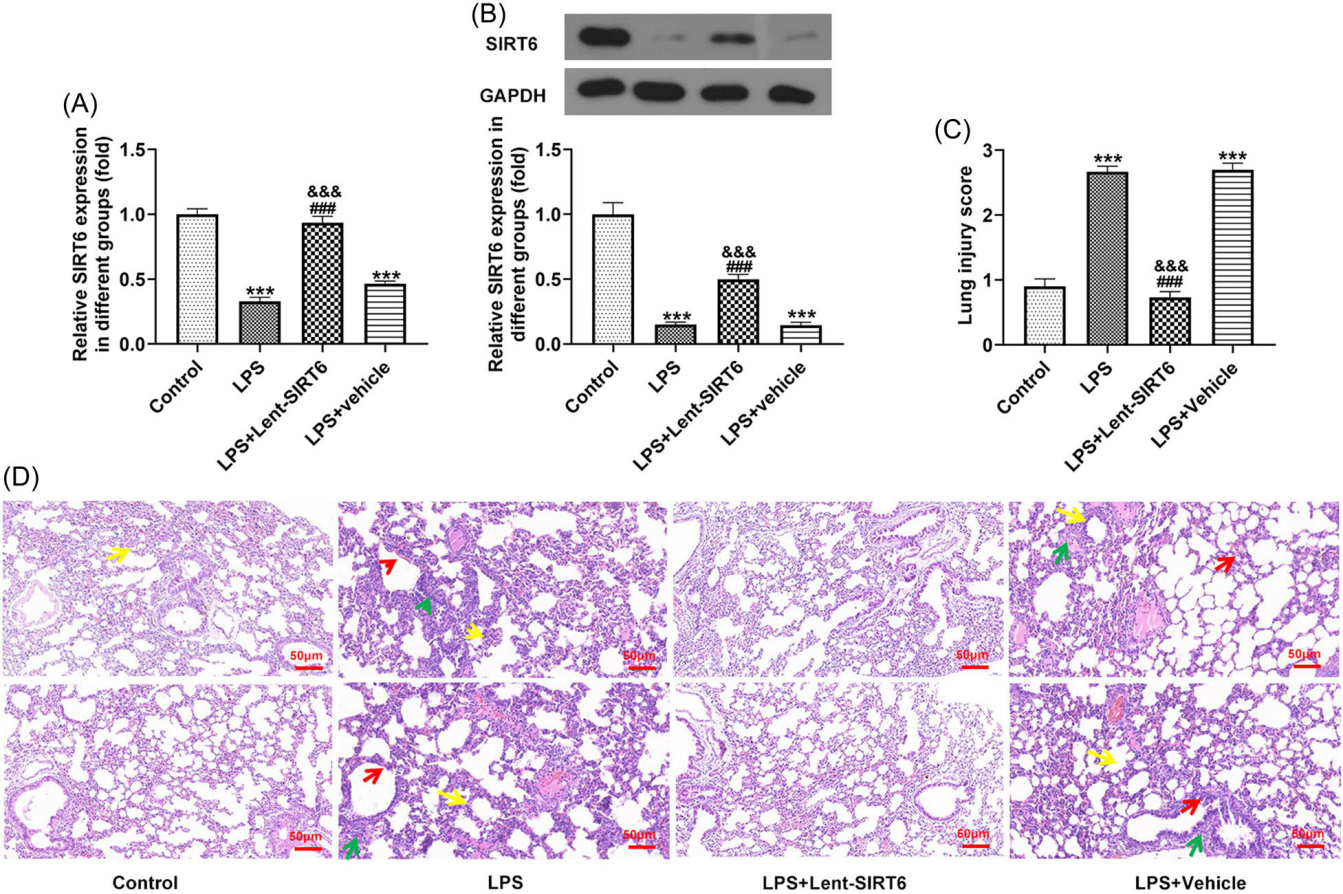
Overexpression of SIRT6 alleviated lung pathological injury in LPS‐induced mice. (A) RT‐qPCR tested SIRT6 mRNA expression in lung tissues after overexpression of SIRT6 in LPS‐induced mice. (B) Western blot tested SIRT6 protein expression in lung tissues after overexpression of SIRT6 in LPS‐induced mice. (C) Pathological score of lung injury. (D). HE staining judged pathological alternations in lung tissues. ***p < .001 vs. Control, ^&&&^ *p* < .001 vs. LPS, ^###^ *p* < .001 vs. LPS + Vehicle. HE, hematoxylin and eosin; LPS, lipopolysaccharide; mRNA, messenger RNA; RT‐qPCR quantitative reverse‐transcription polymerase chain reaction.

First, SIRT6 expression in lung tissues was examined with RT‐qPCR and Western blot. The results manifested that SIRT6 expression was prominently declined in the LPS group relative to the control group. By contrast with LPS + Vehicle group, SIRT6 expression was distinctly fortified in the LPS + Lent‐SIRT6 group (Figure 1A,B). Subsequently, the results from HE staining implied that the pathological state of lung tissue in the LPS group was more serious than that in the control group. SIRT6 expression did not change between LPS and LPS + Vehicle groups. Relative to the LPS and LPS + Vehicle groups, the pathological status of lung tissues was reduced in the LPS + Lent‐SIRT6 group (Figure 1C,D).

### 3.2 Overexpression of SIRT6 reduced pulmonary edema and the proportion of cells in BALF in LPS‐induced mice

**Figure 2:**
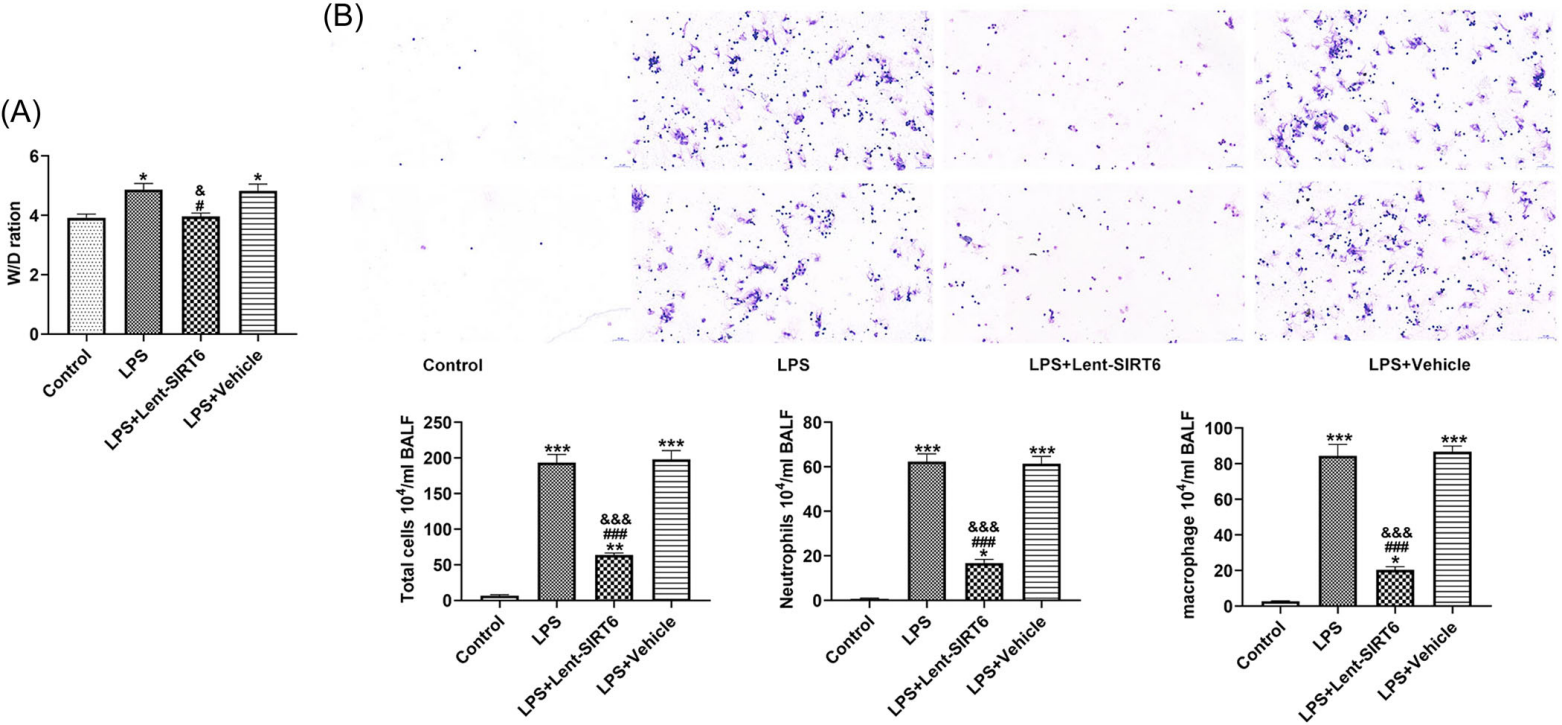
Overexpression of SIRT6 reduced pulmonary edema and the proportion of cells in BALF in LPS‐induced mice. (A) W/D ratio was measured to observe the change of pulmonary edema. (B) Giemsa staining was used to count BALF cell, and neutrophils and macrophages were also counted. *p < .05, ***p < .001 vs. Control; ^&^ *p* < .05, ^&&&^ *p* < .001 vs. LPS; ^#^ *p* < .05, ^###^ *p* < .001 vs. LPS + Vehicle. LPS, lipopolysaccharide.

Through assessment of lung W/D ratio, it was noted that in the LPS group, the lung edema was increased by contrast with the control group. Relative to the LPS and LPS + Vehicle groups, pulmonary edema was reduced in the LPS + Lent‐SIRT6 group (Figure 2A). Through Giemsa staining, we also found that total cell, neutrophil, and macrophage proportion were increased in the LPS group by contrast with the control group. Total cell, neutrophil, and macrophage proportion were decreased in the LPS + Lent‐SIRT6 group by contrast with LPS and LPS + Vehicle groups (Figure 2B).

### 3.3 Overexpression of SIRT6 attenuated inflammatory response and lung cell apoptosis in LPS‐induced mice

**Figure 3:**
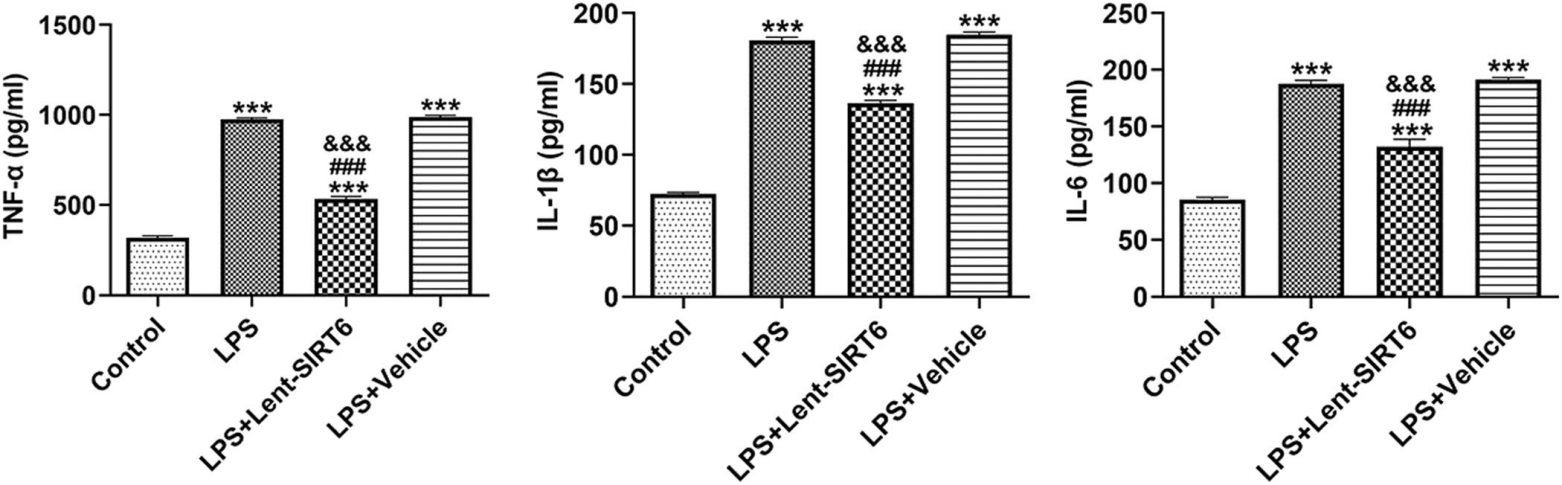
SIRT6 elevation ameliorated inflammatory response in LPS‐insulted mice. ELISA examined TNF‐α, IL‐1β, and IL‐6 activities in the mouse serum. ***p < .001 vs. Control, ^&&&^ *p* < .001 vs. LPS, ^###^ *p* < .001 vs. LPS + Vehicle. ELISA, enzyme‐linked immunosorbent assay; IL, interleukin; LPS, lipopolysaccharide; TNF‐α, tumor necrosis factor‐α.

**Figure 4:**
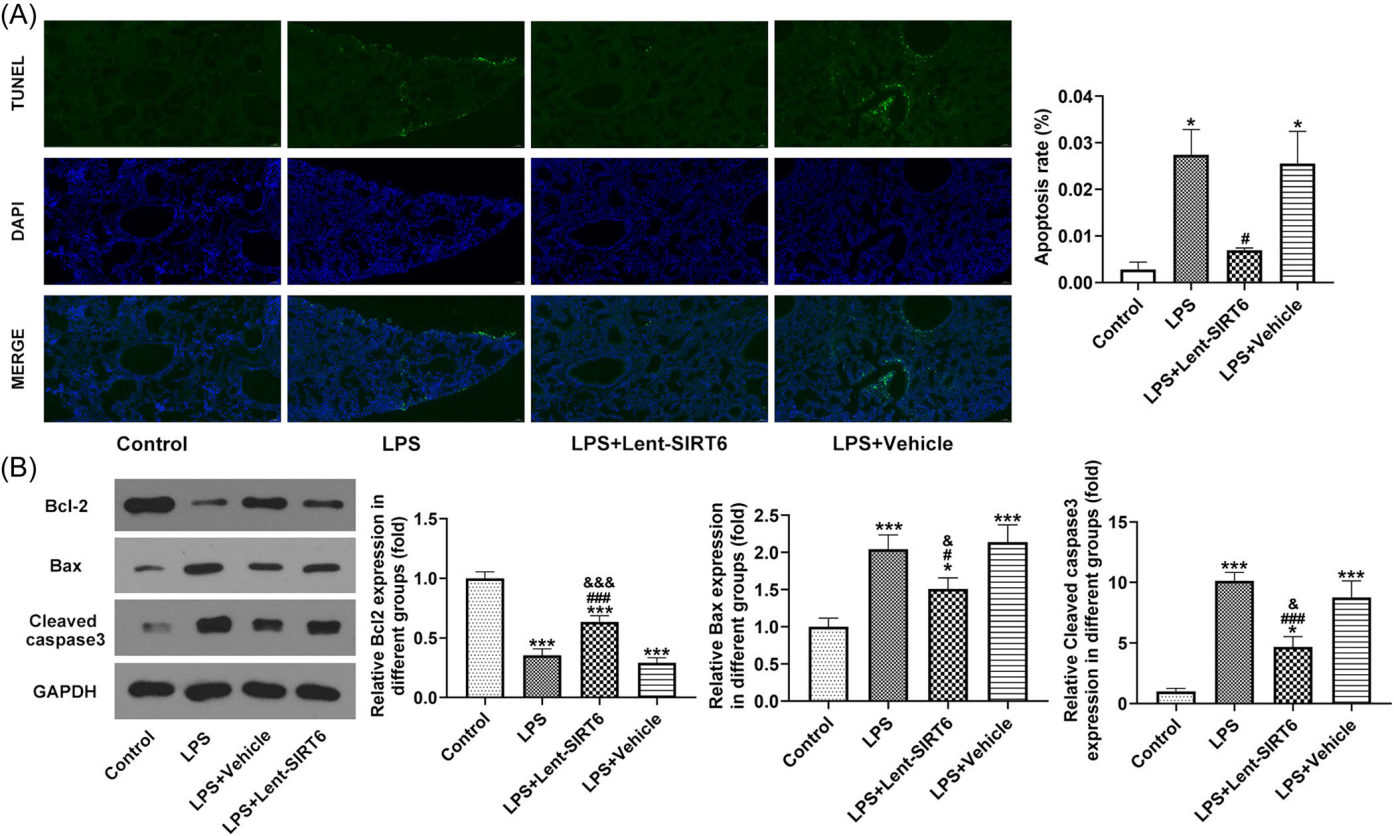
Overexpression of SIRT6 attenuated lung cell apoptosis in LPS‐induced mice. (A) Tunel assay detected the apoptosis of lung cells after overexpression of SIRT6 in LPS‐induced mice. (B) Western blot tested apoptosis‐associated protein Bcl‐2, Bax, and Cleaved caspase3 expression in lung tissues. *p < .05, ***p < .001 vs. Control; ^&^ *p* < .05, ^&&&^ *p* < .001 vs. LPS; ^#^ *p* < .05, ^###^ *p* < .001 vs. LPS + Vehicle. LPS, lipopolysaccharide.

ELISA results elucidated that TNF‐α, IL‐1β, and IL‐6 in the LPS group were notably aggrandized by contrast with the control group. Relative to LPS and LPS + Vehicle groups, TNF‐α, IL‐1β, and IL‐6 expressions were decreased in the LPS + Lent‐SIRT6 group (Figure 3). TUNEL and western blot results illuminated that apoptosis level was elevated in the LPS group, which was also evidenced by increased Bax, Cleaved caspase3 expression, and lessened Bcl2 expression by contrast with the control group. Relative to LPS and LPS + Vehicle groups, the apoptotic cells in the LPS + Lent‐SIRT6 group were decreased, which was evidenced by declined Bax, cleaved caspase3 expression, and augmented Bcl2 expression (Figure 4A,B).

### 3.4 Overexpression of SIRT6 regulated ACE2/STAT3/PIM1 signaling in LPS‐induced mice

**Figure 5:**
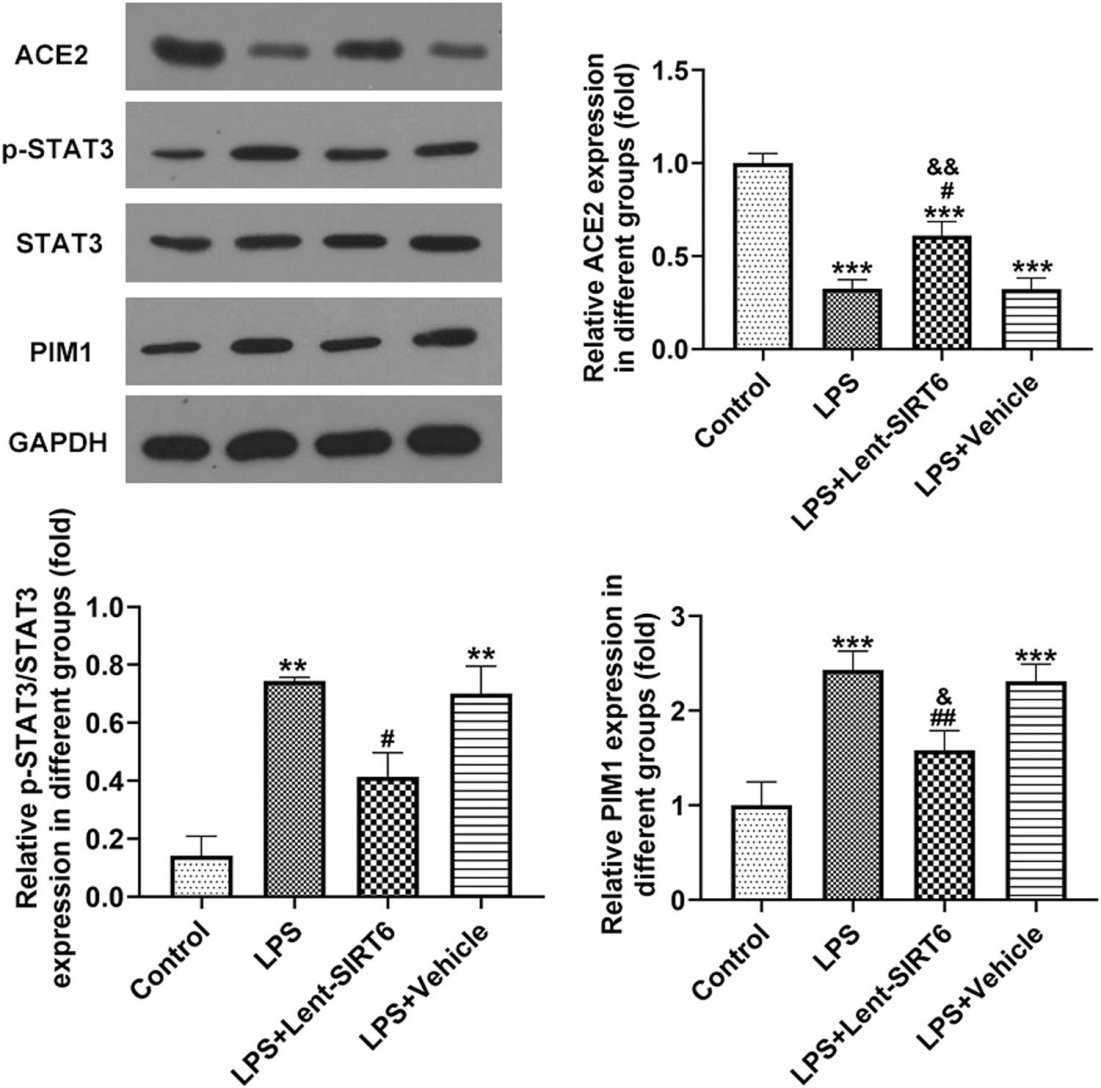
Overexpression of SIRT6 regulated ACE2/STAT3/PIM1 signaling in LPS‐induced mice. Western blot detected the expression of ACE2/STAT3/PIM1 signaling‐related proteins in lung tissues. **p < .01, ***p < .001 vs. Control; ^&^ *p* < .05, ^&&^ *p* < .05, ^&&&^ *p* < .001 vs. LPS; ^#^ *p* < .05, ^#^ *p* < .01 vs. LPS + Vehicle. LPS, lipopolysaccharide.

In the experiment, we found abnormal expression of ACE2/STAT3/PIM1 signal‐associated proteins in lung tissues after overexpression of SIRT6. Western blot results elaborated that p‐STAT3 and PIM1 expression in the LPS group were increased, while ACE2 expression was cut down relative to the control group. p‐STAT3 and PIM1 expression were decreased in LPS + Lent‐SIRT6 group, while ACE2 expression was fortified obviously by contrast with LPS and LPS + Vehicle groups (Figure 5).

### 3.5 The regulation of SIRT6 on ACE2/STAT3/PIM1 signaling in LPS‐induced lung epithelial cells

**Figure 6:**
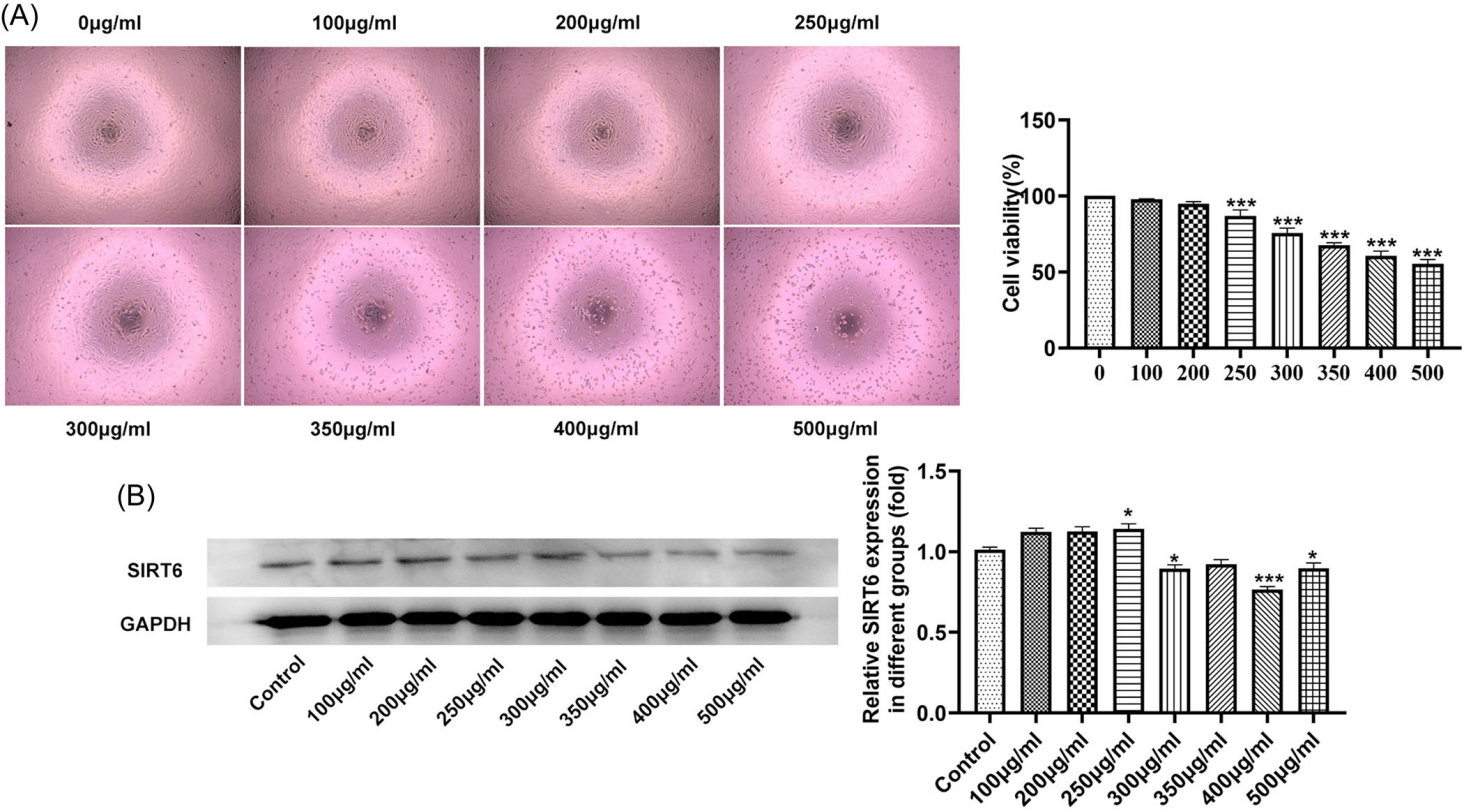
SIRT6 expression in LPS‐treated lung epithelial cells. (A) Lung epithelial cells were induced by different concentrations of LPS and CCK8 appraised cell activity. (B) Western blot tested SIRT6 expression in lung epithelial cells induced by different concentrations of LPS. *p < .05, ***p < .001 vs. control. CCK8, Cell Counting Kit‐8; LPS, lipopolysaccharide.

**Figure 7:**
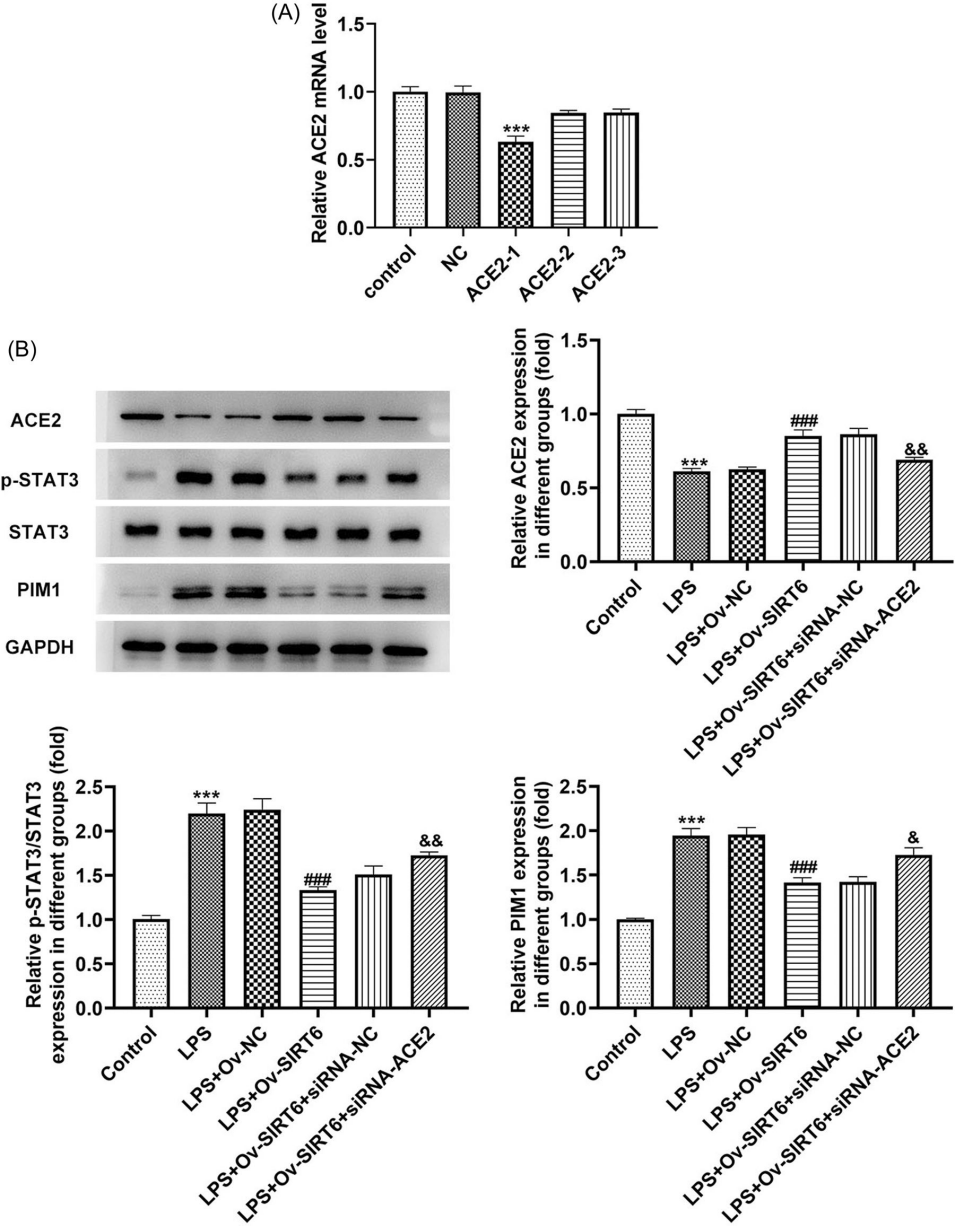
The regulation of SIRT6 on ACE2/STAT3/PIM1 signaling in LPS‐induced lung epithelial cells. (A) RT‐qPCR tested ACE2 expression after the interference plasmid of ACE2 was constructed. ***p < .001 vs. NC. (B) Western blot detected the expression of ACE2/STAT3/PIM1 signaling‐related proteins ACE2, p‐STAT3, STAT3, and PIM1. ***p < .001 vs. control; ^###^ *p* < .001 vs. LPS + Ov‐NC; ^&^ *p* < .05, ^&&^ *p* < .01 vs. LPS + Ov‐SIRT6 + siRNA‐NC. LPS, lipopolysaccharide; RT‐qPCR quantitative reverse‐transcription polymerase chain reaction; siRNA, small interfering RNA.

Next, we will discuss the mechanism in cell experiments. Lung epithelial cells were induced by different concentrations of LPS and cell activity was detected by CCK8. The results showed that LPS treatment induced cell activity injury (Figure 6A). Subsequently, western blot results revealed that SIRT6 expression was notably lessened upon exposure to 300 μg/mL of LPS (Figure 6B). Therefore, 300 μg/mL LPS was adopted for the ensuing experiments. The interference plasmid of ACE2 was constructed, and the plasmid transfection efficacy was detected by RT‐qPCR. The interference efficacy siRNA‐ACE2‐1 was better than others, as shown in Figure 7A. Then Control, LPS, LPS + Ov‐NC, LPS + Ov‐SIRT6, LPS + Ov‐SIRT6 + siRNA‐NC, and LPS + OV‐SIRT6 + siRNA‐ACE2 groups were classified into. Western blot results uncovered that p‐STAT3 and PIM1 expression were augmented in the LPS group, while ACE2 expression was declined relative to the control group. p‐STAT3 and PIM1 expressions were cut down, and ACE2 expression was aggrandized in LPS + Ov‐SIRT6 group relative to LPS + Ov‐NC group. p‐STAT3 and PIM1 expression in LPS + OV‐SIRT6 + siRNA‐ACE2 group were increased but were lower than that in the LPS group, while ACE2 expression was decreased but was higher than that in the LPS group relative to LPS + Ov‐SIRT6 + siRNA‐NC group (Figure 7B).

### 3.6 SIRT6 attenuated inflammatory response and apoptosis of LPS‐induced lung epithelial cells through ACE2/STAT3/PIM1 signaling

**Figure 8:**
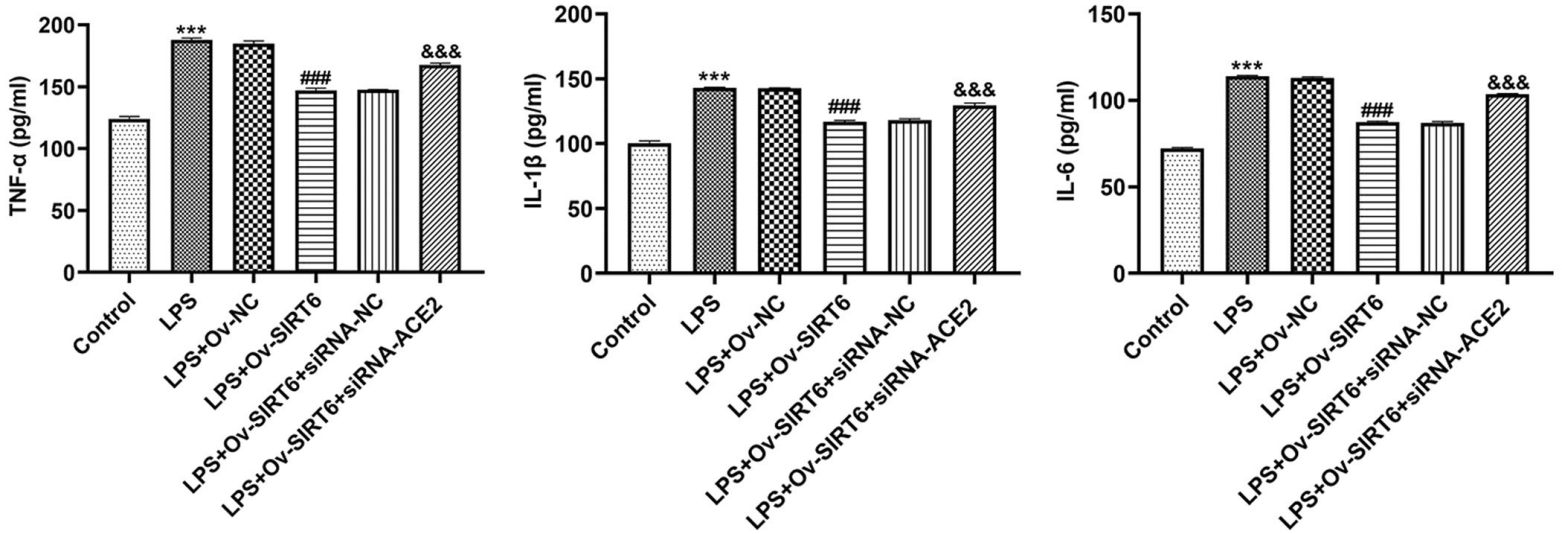
SIRT6 attenuated inflammatory response of LPS‐induced lung epithelial cells through ACE2/STAT3/PIM1 signaling. ELISA examined TNF‐α, IL‐1β, and IL‐6 activities in the culture medium of lung epithelial cells. ***p < .001 vs. control; ^###^ *p* < .001 vs. LPS + Ov‐NC; ^&&&^ *p* < .001 vs. LPS + Ov‐SIRT6 + siRNA‐NC. ELISA, enzyme‐linked immunosorbent assay; IL, interleukin; LPS, lipopolysaccharide; TNF‐α, tumor necrosis factor‐α; siRNA, small interfering RNA.

**Figure 9:**
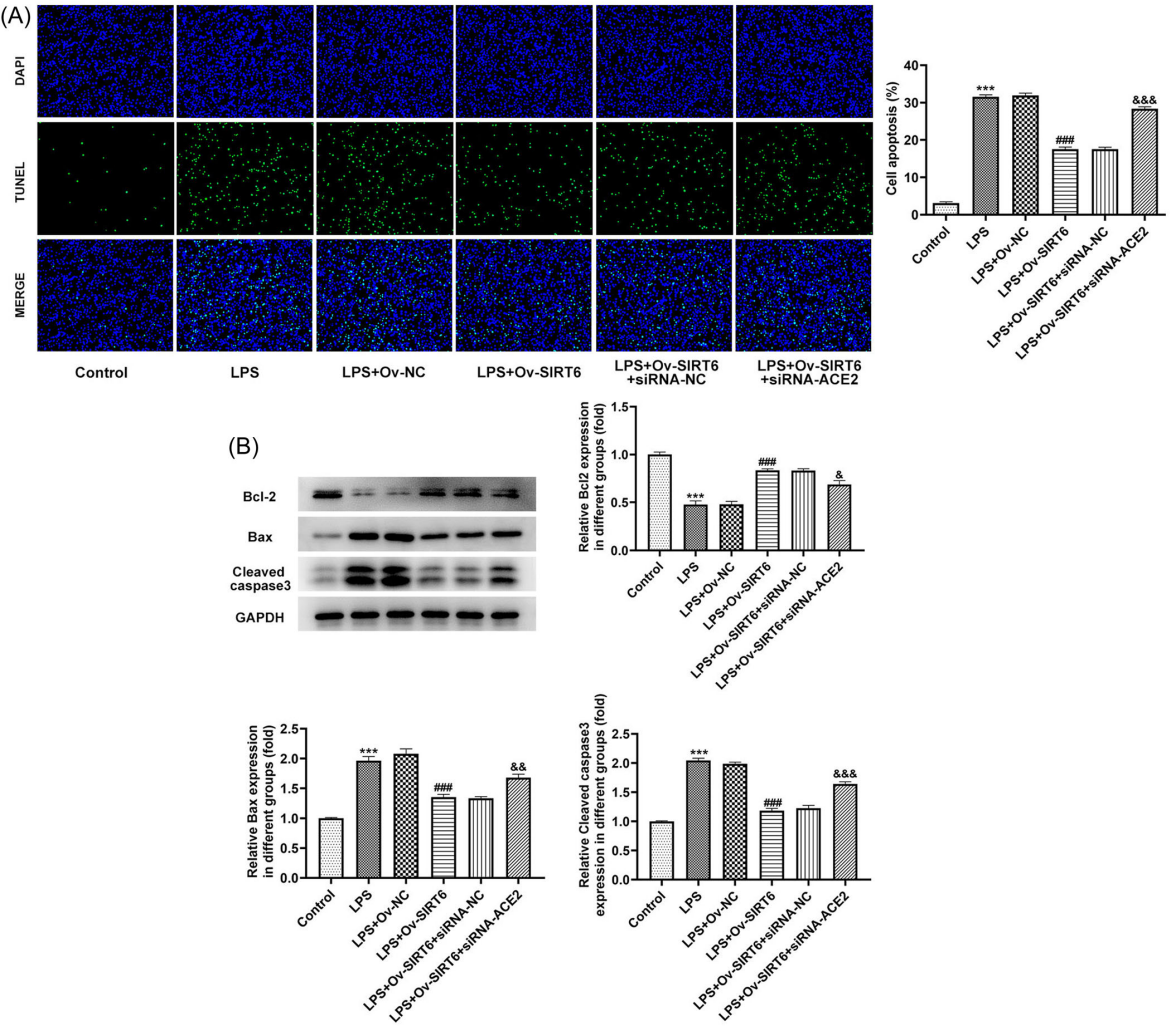
SIRT6 attenuated apoptosis of LPS‐induced lung epithelial cells through ACE2/STAT3/PIM1 signaling. (A) Tunel assay detected the apoptosis of lung epithelial cells after SIRT6 and ACE2 were transfected simultaneously. (B) Western blot tested apoptosis‐associated protein Bcl‐2, Bax, and Cleaved caspase3 expression in lung epithelial cells. ***p < .001 vs. control; ^###^ *p* < .001 vs. LPS + Ov‐NC; ^&^ *p* < .05, ^&&^ *p* < .01, ^&&&^ *p* < .001 vs. LPS + Ov‐SIRT6 + siRNA‐NC. LPS, lipopolysaccharide.

ELISA was to test contents of TNF‐α, IL‐1β, and IL‐6 in the culture medium of lung epithelial cells. It turned out that TNF‐α, IL‐1β, and IL‐6 expression in the LPS group were elevated by contrast with the control group. TNF‐α, IL‐1β, and IL‐6 expression were markedly lessened in LPS + Ov‐SIRT6 group by contrast with LPS + Ov‐NC group. After further inhibition of ACE2 expression, in LPS + Ov‐SIRT6 + siRNA‐ACE2 group, TNF‐α, IL‐1β, and IL‐6 expression were increased, whereas lower than in the LPS group (Figure 8). Similarly, by contrast with the control group, apoptosis level was boosted in the LPS group, evidenced by fortified Bax, Cleaved caspase3 expression, and declined Bcl2 expression. The apoptosis level of the LPS + Ov‐SIRT6 group was attenuated, Bax and Cleaved caspase3 expressions were reduced, and Bcl2 expression was increased by contrast with the LPS + Ov‐NC group. Relative to the LPS + Ov‐SIRT6 + siRNA‐NC group, the level of apoptotic cells in the LPS + Ov‐SIRT6 + siRNA‐ACE2 group was increased, whereas lower than that in the LPS group, and Bax, Cleaved caspase3 expression levels were increased but lower than those in the LPS group, and Bcl2 expression levels were decreased but higher than those in the LPS group (Figure 9A,B).

## 4 DISCUSSION

LPS is widely recognized as a lung injury inducer and has been applied in ALI model construction.^23^, ^24^ Direct injection of LPS into lung tissues may result in increased accumulation of peripheral inflammatory cells, increased pulmonary capillary permeability, alveolar and interstitial edema, and eventually lead to acute inflammatory response.^25^ In this study, we induced mice and lung epithelial cells with LPS in vitro and in vivo to establish ALI models. We found that the pathological state of the lungs of mice induced by LPS was severe, with edema in the lungs and enhanced proportion of total cells, neutrophils, and macrophages, as well as inflammation and apoptosis in the lung tissues and lung epithelial cells, suggesting the successful construction of lung injury model. LPS plays a key role in the apoptosis of pulmonary endothelial and epithelial cells,^26^, ^27^ and pulmonary epithelial cell apoptosis is one of the key factors in the formation of blood–brain barrier in pulmonary fibrosis.^28^ Therefore, it is of great significance to effectively inhibit the inflammation and apoptosis of lung epithelial cells in ALI.

It was discovered that SIRT6 expression was remarkably reduced in LPS‐induced animals and cells. SIRT6 is a histidine deacetylase that plays a role in injury‐related diseases induced by inflammation.^29^, ^30^ A previous study has showed that SIRT6 has a synergistic effect on SIRT1.^31^ And a number of studies have reported that SIRT1 has a protective effect on various types of lung injury, LPS‐induced lung injury is also included.^9^, ^10^ In our experiment, it was found that overexpression of SIRT6 could significantly improve the pathological state and pulmonary edema of LPS‐induced mice, and inhibit pulmonary inflammation and pulmonary cell apoptosis, which implied that overexpression of SIRT6 could improve the symptoms of ALI disease. Studies have shown that SIRT6 eases sepsis‐elicited acute respiratory distress syndrome via promoting macrophage M2 polarization.^32^ Moreover, in sepsis‐evoked ALI, SIRT6 positively regulates Nrf2 expression, activates Nrf2‐mediated anti‐inflammatory and antioxidant enzymes, and can effectively alleviate LPS‐induced inflammation of HUVECs.^7^ These reports were in agreement with our experimental results.

**Figure 10:**
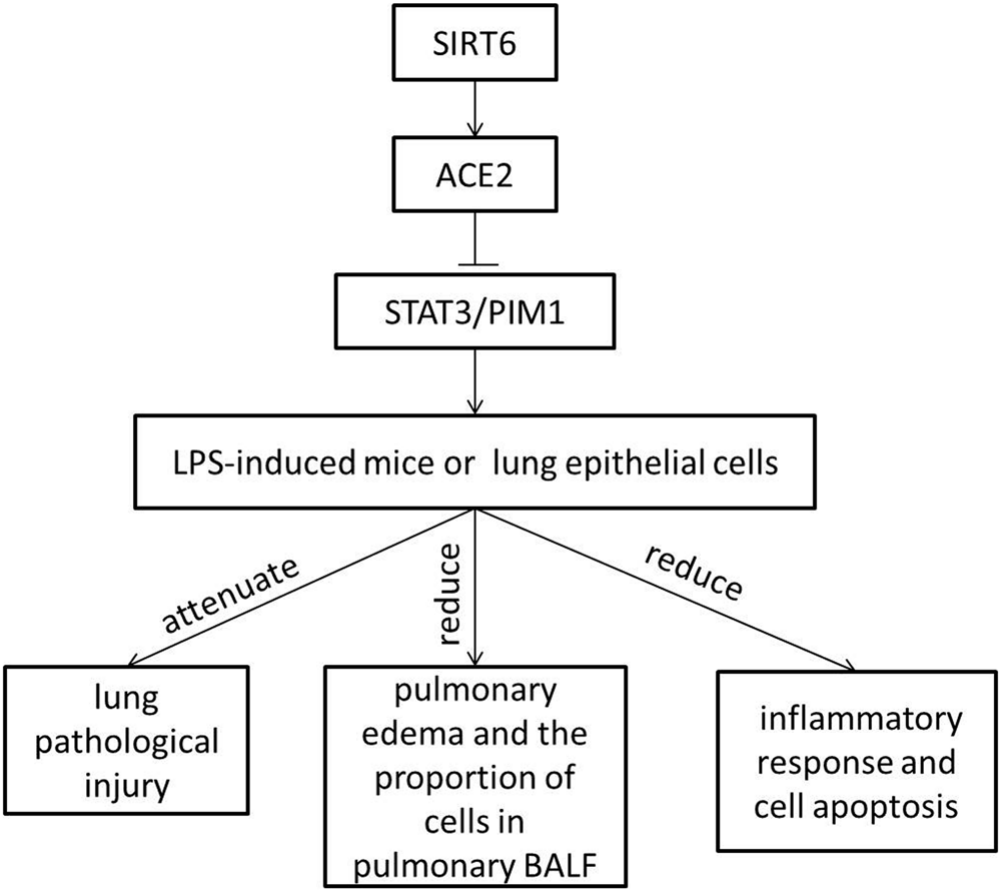
The specific mechanism diagram. LPS, lipopolysaccharide.

The study has shown that ACE2 has a protective effect on LPS‐induced ALI in mice by inhibiting LPS–TLR4 pathway.^15^ Severe acute respiratory syndrome coronavirus receptor ACE2 protects lungs from damage.^33^ It has been shown that SIRT6‐ACE2 has a regulatory effect, and SIRT6 can regulate the expression of ACE2 in rat aortic membrane fibroblasts.^14^ In our experiment, it was found that when SIRT6 was upregulated, the expression of ACE2 in mouse lung tissues and lung epithelial cells was significantly increased. Therefore, we hypothesized that SIRT6 played a role in ALI by regulating ACE2. Moreover, ACE2 is upregulated by IL‐6 through STAT3 signaling in synovial tissues.^34^ KEGG Pathway showed that STAT3 activation could activate the expression of downstream target gene PIM1. In addition, PIM1 targeting can reduce apoptosis and oxidative stress in CCL4‐induced ALI in mice.^35^ In this experiment, we found that the expression of p‐STAT3 and PIM1 in cells was significantly inhibited after SIRT6 expression was knocked down. At this point, the inflammatory response and apoptosis were suppressed. Inhibition of ACE2 could reverse the inhibitory effect of SIRT6 overexpression on downstream STAT3 and PIM1, and reverse the protective effect of SIRT6 overexpression on cells. See Figure 10 for the specific mechanism diagram.

Our article also has some limitations. In the process of mechanism study, we only inhibited the expression of ACE2, but no experiments have been conducted to verify ACE2 overexpression. In the following experiments, we will further overexpress ACE2 so as to explore the regulatory mechanism of SIRT6.

## 5 CONCLUSION

To sum up, we found that SIRT6 mitigated LPS‐elicited inflammation and apoptosis of lung epithelial cells in ALI through ACE2/STAT3/PIM1 signaling. Our paper offered a solid theoretical basis for the therapy for sepsis‐induced ALI.

## AUTHOR CONTRIBUTIONS

Juan Yang and Xing Chen performed the research. Xing Chen designed the research study. Juan Yang contributed essential reagents or tools. Xing Chen analysed the data. Juan Yang and Xing Chen wrote the paper. All authors have read and approved the final manuscript.

## CONFLICTS OF INTEREST STATEMENT

The authors declare no conflicts of interest.

## ETHICS STATEMENT

All animal procedures were operated in light of the NIH Guide for the Care and Use of Laboratory Animals, approved by the ethical guidelines of Shandong Provincial Hospital, Cheeloo College of Medicine, Shandong University (approve number: NO.2022‐016), and were conducted in light of the ARRIVE guidelines.

## CONSENT FOR PUBLICATION

All the authors agreed to be published.

## Data Availability

The analyzed data sets generated during the present study are available from the corresponding author on reasonable request.
